# Characterization of Ex Vivo Expanded Oral Mucosal Epithelium Cells on Acellular Porcine Corneal Stroma for Ocular Surface Reconstruction

**DOI:** 10.1155/2017/6761714

**Published:** 2017-05-08

**Authors:** Jia-Song Wang, Hua-Tao Xie, Ming-Chang Zhang

**Affiliations:** Department of Ophthalmology, Union Hospital, Tongji Medical College, Huazhong University of Science and Technology, Wuhan 430022, China

## Abstract

**Purpose:**

To ex vivo expand oral mucosal epithelium cells (OMECs) on acellular porcine corneal stroma (APCS) without using feeder cells and serum and to compare the morphologic and phenotypic characteristics of cultured oral cells on APCS to those of cells on deluded human amniotic membrane (HAM).

**Methods:**

SD rat oral mucosal biopsies were cultured on APCS and HAM. Reverse-transcription polymerase chain reaction (RT-PCR) and immunohistochemistry were used to analyze the characterization of stem cells and epithelial differentiation of the outgrowth products.

**Results:**

Stratified and optimal transplantable OMECs were obtained after being cultured three to four weeks. Both RT-PCR and immunohistochemistry showed that cultured OMECs expressed markers of epithelial differentiation cytokeratin K3 and epithelial stem cell markers of p63 and ABCG2.

**Conclusions:**

OMECs can be successfully cultured on APCS without using xenobiotic feeder cells and serum. Characterization showed that these sheets retain the morphologic and phenotypic characteristics of OMECs within differentiated cells and stem cells. The optimal transplantable sheets can prove to be particularly beneficial to both bilateral limbal stem cell deficiency and deep corneal lesions.

## 1. Introduction

Limbal stem cells (LSCs) have been proven with two main functions: renewed corneal epithelial cells and conjunctival and blood vessel ingrowth onto the cornea. Although located at the basal layer of the limbal epithelium [1, 2], it also could be fully destroyed by severe ocular surface diseases (OSD), including chemical burns, microbiological infections, autoimmune diseases, and genetic disorders, which finally results in limbal stem cell deficiency (LSCD). As a result, corneal inflammation, neovascularization, conjunctivalization, ocular pain, scarring, deep corneal cloudiness, and loss of vision appeared. LSCD is divided into two categories according to the eyes suffering [3–5]: unilateral LSCD, for which autologous LSCs transplantation is preferred [6–11], and a more challenging bilateral LSCD. Recently, a number of studies have reported cultivated oral mucosal epithelial cell (COMECs) transplantation for bilateral LSCD with several advantages including high proliferative potential, stability of a long time without keratinization [12–16], and high transplantation success rate [17–25]. However, previously, COMECs were mainly used in superficial corneal diseases, such as corneas covered by a thin vascular membrane, with or without the substrate and carrier such as contact lenses (CLs) [26] and human amniotic membrane (HAM) [10, 27–35]. If patients have both LSCD and deep corneal cloudiness, keratoplasty should be followed by the COMECs transplantation. Shortages of donor corneas remain a challenge worldwide, especially in Asian countries such as China [36]. Acellular porcine corneal stroma (APCS) grafts should be a good substitute for lamellar keratoplasty [37–40]. APCS with a mesh structure has a good cellular affinity and can relatively provide not only a healthy but also a stable corneal stroma microenvironment as well as a substrate that is advantageous to stem cells and epithelial cell adhesion and growth. However, to our knowledge, the ex vivo expansion of oral mucosal epithelial cells (OMECs) on APCS has never been reported previously. In this study, we aimed to establish a method of COMECs on APCS that could be used for the ocular surface reconstruction with both LSCD and deep corneal cloudiness.

## 2. Methods

### 2.1. Preparation of APCS and HAM

HAM were prepared using a previously described method [41, 42]. In short, according to the Declaration of Helsinki and written informed consent, HIV, HPV B and C, and syphilis were excluded by serologic tests and the placentas were harvested after cesarean deliveries immediately. The HAM was separated from the chorion after washing 3 times using saline containing 50 g/ml penicillin, 50 g/ml streptomycin, and 2.5 g/ml amphotericin B and then cut into 4 × 4 cm pieces preserved in sterilized pure glycerin (Wuhan Union Hospital) at −20°C. Before using, the membranes were thawed, washed off glycerin, and then rehydrated in saline for 10 minutes. Then, the HAM was treated with 0.25% trypsin-EDTA at 37°C for 30 minutes to denude the epithelial lining.

APCS were supported by the ZhongHao Corneal Engineering Corporation (Qingdao, China) according to the method previously reported [43]. Briefly, pig eyes were obtained from a quarantined animal facility certified by the Bureau of Animal Quarantine Department of China. The eyes were enucleated immediately after death and thoroughly washed 3 times with phosphate-buffered saline (PBS), and whole corneas were cut from the eyes. The corneal stroma then underwent agitation in 2 M NaCl for 30 min, followed by ultrapure water for 30 min. The process was repeated three times. Next, 0.2% Triton X-100 was used to wash the corneas for 6 h. After a thorough washing in PBS, the APCSs were dehydrated in glycerol to the normal thickness of a native cornea. Finally, sterilization was performed by Co60 irradiation. Furthermore, make sure the prepared APCS passed cytotoxicity and histocompatibility tests. Before use, APCS were rehydrated in saline for 10 minutes.

### 2.2. Harvesting of Oral Mucosal Epithelial Tissue

SD rats weighing 150–200 g were subjected to a protocol approved by the Animal Research Committee of the Huazhong University of Science and Technology. The buccal oral mucosa biopsy specimens, each 2 to 4 mm^2^ in size, were obtained from rats with anesthesia by intramuscular injection of xylazine hydrochloride (5 mg/mL) and ketamine hydrochloride (50 mg/mL). The connective tissue was removed using dissecting scissors, and then the harvested tissue was washed three times (3 min each time) with Dulbecco's phosphate-buffered saline (PBS, Sigma-Aldrich) containing antibiotics and amphotericin. These tissues were then incubated at 37°C for 1 hour with Dispase II (Roche Diagnostics GmbH, Mannheim, Germany) to separate the epithelium from the remaining connective tissue and then cut into 1 mm^2^ pieces (explanted tissue) under sterile conditions.

### 2.3. COMECs (Explanted Technique)

**Figure 1:**
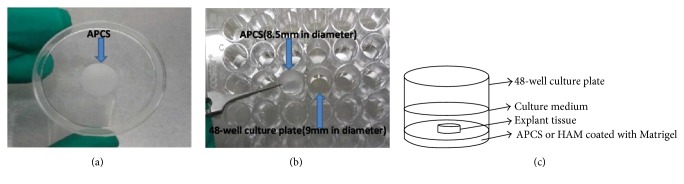
(a), (b) 48-well plate with 9 mm well diameter and APCS with 8.5 mm in diameter. (c) Explant tissue culture pattern.

Firstly, APCS were immersed in PBS containing 5% Matrigel™ (Becton Dickinson) in 48-well culture plates for one hour at 37°C. Secondly, the explanted tissue was placed on APCS and adhered to APCS for 1 to2 hours before addition of media and then incubated at 37°C with 5% CO_2_. All cultures were maintained with growth medium containing Keratinocyte Serum Free Medium (Keratinocyte-SFM, Life Technologies Corporation, Carlsbad, CA) supplemented with 5 ng/ml of h-EGF and changed every other day (Figure 1). The outgrowth stage was recorded under a phase-contrast inverted microscope. The area of explant outgrowth was marked on the underside of the culture well at the time of each feed until APCS was covered completely with multilayer epithelium cells and suitable for a graft.

Meanwhile, the explanted tissue was placed on de-epithelialized human amniotic membrane (HAM) and cultured with the same method as a control group.

### 2.4. Cytopathology of COMECs

COMECs sheets were mechanically detached from APCS and HAM scaffold using a sterile cell scraper and tissue forceps. The sheets were washed with 0.1 M phosphate buffer (pH 7.4), fixed in a 3 : 1 acetic acid-methanol mixture, and spread on a polylysine-coated glass slide. Multiple slides of a single specimen were stained with hematoxylin-eosin (H&E).

### 2.5. Reverse-Transcription Polymerase Chain Reaction (RT-PCR)

Total RNA was extracted from the sheets of COMECs after mechanically detaching them from APCS or HAM using a sterile cell scraper, using 1 ml reagent (Tri-Reagent; Aidlab) in biological replicates. Then, RNA was treated with DNase, and RNA was used for cDNA synthesis using HiScript reverse transcriptase (RNase H; Genecopoeia). Glyceraldehyde-3-phosphate dehydrogenase (GAPDH) primers were used to confirm the integrity of the cDNA. The mixture for the PCR amplification reactions was denatured at 95°C for 10 minutes, followed by 40 cycles at 95°C for 30 seconds and 60°C for 30 seconds. A polymerase chain reaction (PCR) was performed on this cDNA using the primers shown in Table 1, and the PCR products were separated on a 1.2% agarose gel.

### 2.6. Immunocytochemical Analysis

Target proteins were assessed by immunohistochemistry in the COMECs sheet. The paraffin-sectioned slides were fixed in 10% neutral buffered formalin. Then, sections or trypsin-EDTA (Sigma-Aldrich) digested into single cells were incubated overnight at 4°C with primary antibodies against cytokeratin K3/K12, AE5 clone (1 : 150 dilution) (Chemicon, Billerica, MA), p63 with p63 4A4 clone (1 : 150 dilution) (Millipore, Billerica, MA), and ABCG2 with Abcam (1 : 150 dilution) (Cambridge, MA), separately. Anti-rabbit/mouse/goat (1 : 100 dilution) as the secondary antibodies and DAPI for staining nuclei was also used. Pictures were observed using a light microscope.

Meanwhile, OME was digested with 0.05% trypsin, a single-cell suspension resuspended in medium at a density of 1 × 10^6^ cells per square centimeter. And cells were spun on glass slides by cytospin, dried at RT, fixed in methanol, and then stained (as mentioned above). Then, the percentage of positive cells was calculated based on the average of positive cells in a total of 100 cells decided by two independent researchers.

### 2.7. Statistical Analysis

Data were showed as mean ± SD. Student's *t*-test was used to compare the two groups. A *p* value of <0.05 was considered significant.

## 3. Results

To investigate the feasibility of APCS as a carrier for ex vivo OMECs growth, we observed the growth of cells and designed RT-PCR and immunocytochemistry to explore the morphology and phenotype of COMECs.

### 3.1. Morphologic Characterization of COMECs

**Figure 2:**
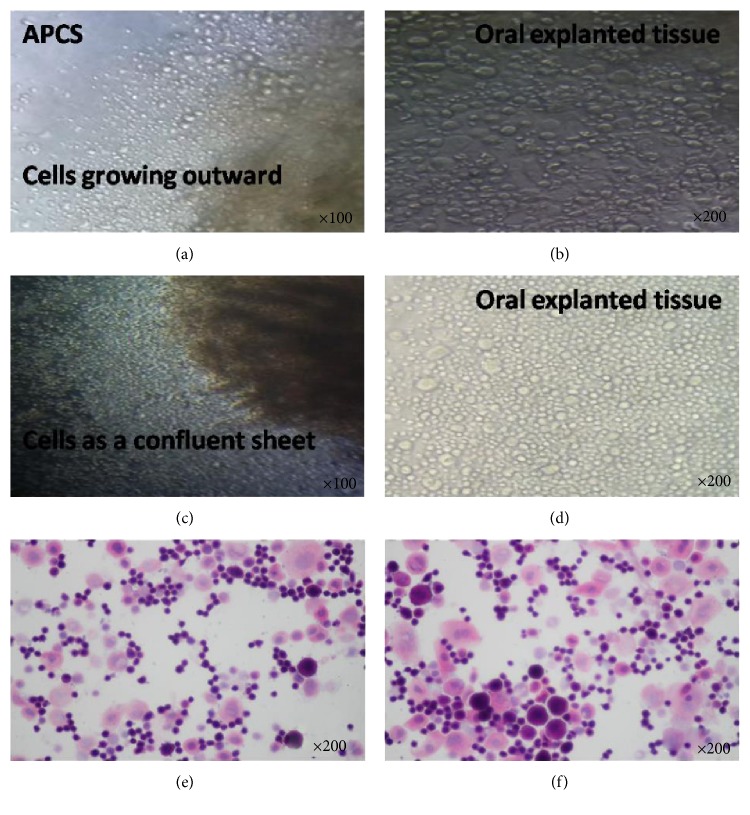
(a), (b) Morphologic finding of OME cells growing on APCS for 2-3 days. Magnification: (a) ×100; (b) ×200. (c), (d) OME cells as a confluent sheet on APCS (after 1-2 weeks). Magnification: (c) ×100; (d) ×200. (e) OMEC (on APCS) (after 2 weeks) stained with H&E (magnification, ×200). (f) OMEC (on HAM) (after 2 weeks); stained with H&E (magnification, ×200).

Cell proliferation and migration initiated from expanded edge was first seen at day 2.4 ± 0.51 (range of 2-3, *n* = 5) (Figures 2(a) and 2(b)), and a multilayered confluent sheet of COMECs appeared, which exhibited a confluent cobblestone and monolayer which was well attached on APCS within about 2 weeks without air-lifting (Figures 2(c) and 2(d)). A healthy and optimal transplantable sheet was produced in about 21.4 days ± 1.82 (range of 20–25, *n* = 5), whereas compared to APCS, cell growth initiation was faster on HAM. Small-cell colonies on HAM were first observed within 2.2 days ± 0.14 days (range: 1–3, *n* = 5), and a transplantable OMECs sheet was obtained after culturing at day 18.4 ± 2.70 (range: 14–21, *n* = 5), approximately. We also calculated the total cell number of confluent sheets on both the APCS and HAM, and there was no statistically significant difference (*p* > 0.05; *n* = 5) between APCS or HAM (7.2 ± 1.1 × 10^5^ versus 7.7 ± 0.9 × 10^5^).

On H&E staining, most of the COMECs on APCS as well as on HAM seem to be small oval-shaped cells with comparatively larger nuclei, some of which are large irregular polygonal cells with relatively smaller nuclei (Figures 2(e) and 2(f)).

### 3.2. Molecular Phenotype Characterization of COMECs

**Figure 3:**
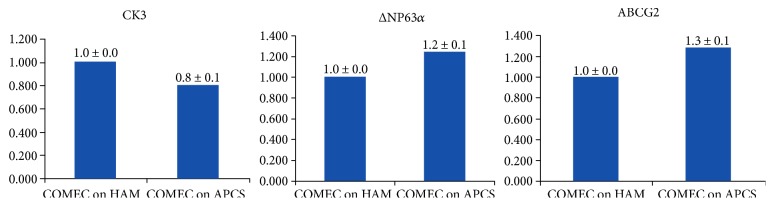
Quantitative real-time PCR analysis of comparison of putative epithelial cell marker expression. Bar chart showing the expression of CK3, ΔNP63*α*, and ABCG2 in oral epithelial cells COME on HAM versus APCS (*P* > 0.05, *n* = 5).

**Figure 4:**
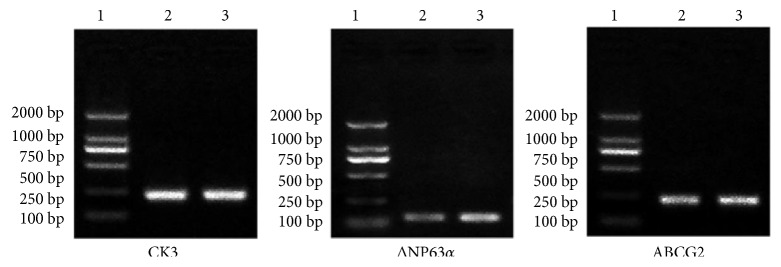
Quantitative real-time PCR analyses of different genes in OMECs isolated from confluent sheets of OMEC cultivated on APCS and HAM. 1, marker genes with different product sizes; 2, COMECs on HAM; 3, COMECs on APCS.

To prove the existence of markers within our COMECs, putative epithelial stem cell markers, namely ΔP63*α* [44, 45] andABCG2 [46, 47], and a specific differentiation marker, cytokeratin K3 (CK3) [14, 48], were firstly examined by RT-PCR. Both of the two groups of COMECs were found to express a corneal and oral epithelial cell marker (CK3) and stem cell markers (ΔNP63*α* and ABCG2) (Figures 3 and 4). RT-PCR analysis also showed higher expression of putative epithelial stem cell markers (ΔNP63*α* and AGCG2) in cultures on APCS when compared with those on HAM and lower quantities of corneal and oral epithelial cell marker (CK3) than those on the HAM group (Figure 3). It seems that COMECs on APCS could remain OMEC stem cell properties. However, there is no statistical significance between the two groups of each marker (*P* > 0.05, *n* = 5).

**Figure 5:**
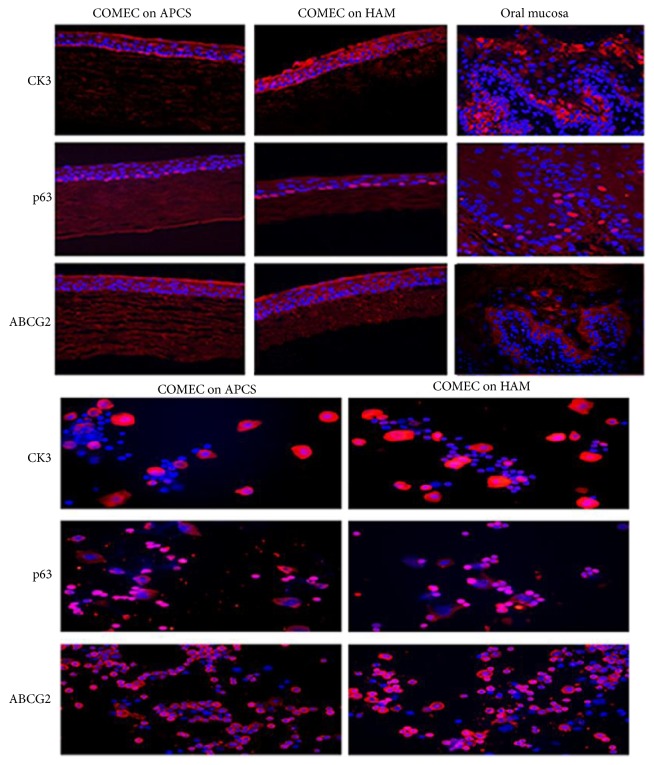
Immunohistochemistry of ex vivo expanded oral epithelium cultured from an explanted culture on either APCS or HAM. Multilayered confluent sheet of COMECs appeared after being cultured three to four weeks, which are well attached on APCS and HAM. It shows the expression of p63 in the nuclei of the basal epithelial sheet and ABCG2 and CK3 expression in the cytoplasm in the full-thickness in cultivated oral mucosal epithelial cell sheets both on APCS or HAM. But ABCG2 and p63 are expressed in the basal layer and CK3 is expressed in full-thickness in rat oral mucosal epithelial cells.

Then immunofluorescence study of the cultured cells was used to confirm RT-PCR results (Figure 5). Both of them generated a five- to seven-layer well-structured epithelium and attached well to the underlying carrier. Regular and tight small cuboidal cells were packed of the basal layer, which were with prominent nuclei that strongly express the putative epithelial stem cell marker p63 and cytoplasmic putative stem cell marker ABCG2. In addition, CK3-positive expression on APCS (40.22 ± 1.23%) was lower than that on HAM (47.06 ± 1.78%) (*p* > 0.05; *n* = 5 in each group). p63-positive expression on APCS and HAM was 57.28 ± 3.35% and 45.65 ± 3.76%, respectively (*p* > 0.05; *n* = 5 in each group). Expression of ABCG2 on APCS and HAM was 38.40 ± 4.96% and 30.58 ± 2.71%, separately (*p* > 0.05; *n* = 5 in each group).

In summary, both of RT-PCR and immunocytochemistry indicated that COMECs could express molecular markers (CK3, p63, ABCG2) identical on APCS and HAM.

## 4. Discussion

In our study, we successfully produced functional COMECs on APCS using the feeder-free and serum-free explanted technique. Sheets that were transparent, with good toughness, with different thickness, and optimally transplantable were produced after about three to four weeks. Morphology and phenotype of the COMECs indicate that the cultures not only keep cells' growth cycle but also express markers of epithelial cells as well as stem cells. The main advantage of this research is the absence of using xenobiotic feeder cells and serum, which has a high risk of transmitting murine-derived diseases to humans, and the main advantage of the new product is that it can be used in deep corneal keratopathy. To our knowledge, this is the first experiment on this subject in the world. These oral epithelial cultures will stand a good chance of being used for ocular surface reconstruction in patients with bilateral LSCD and deep corneal cloudiness.

Murine 3T3 cells have been used in many OME expansion studies, previously [17–23]. COMECs that are absent of xenobiotic feeder cells such as murine 3T3 feeder layers have the advantage of avoiding ethical and pathogen transmissions and providing an unlimited cell source as an alternative therapy for reconstruction of ocular surfaces in patients with bilateral LSCD. For this reason, in recent years, COMECs procedures that are feeder cell-free have dominated in terms of studies, including temperature-responsive cell culture wells [22, 49], fibrin-coated culture inserts [18], laminin-coated CLs [26], cultures coated with Matrigel [50], and HAM [10, 27–35]. Nowadays, more and more researches developed techniques for the COMECS protocol with the use of xenobiotic feeder-free as well as serum-free culture systems [51, 52]. Our technique also has overcome the need of feeder cells and serum while retaining the advantages of the stem cells.

We compared the morphological and phenotypic characteristics of COMECs between the two groups. As mentioned above, HAM are the most common culture substrate and its excellent effect has been proved [10, 20, 27–35, 51–55]. The results of this study demonstrate the success of COMECs on APCS and HAM, in which after 21.4 days ± 1.82 days and 18.4 days ± 2.70 days, respectively, a cultured transplantable OMEC sheet was produced. Obviously, COMECs on HAM outgrew from the edge of explants faster than on APCS, maybe because HAM is more smooth. The mesh structure of APCS can provide space for epithelial cell adhesion, but it is hard to outgrow and crawl. Our studies also indicate that both of the sheets express markers of stratified epithelia CK3 and the progenitor cell marker p63 and the putative stem cell marker ABCG2. Our immunohistochemistry also shows the expression of p63 in the nuclei of the basal epithelial sheet and ABCG2 and CK3 expression in the cytoplasm in the full-thickness in cultivated oral mucosal epithelial cell sheets both on APCS or HAM. Nakamura et al. [24] and Hayashida et al. [49] had reported that the cultured oral epithelial cells thus maintain their original phenotype. Our results are consistent with theirs. Meanwhile, RT-PCR has shown that there are no statistical significance between the two groups of each marker (*P* > 0.05, *n* = 5). So, we can announce that COMECs on APCS could also retain putative epithelial progenitor and stem cells.

In addition, authors would like to emphasize the importance of using APCS as a scaffold, which with different thickness range from 200 *μ*m to 500 *μ*m. There is no need to worry about the deepness of pathological tissues. COMEC on APCS could transplant as a whole, which could solve not only LSCD but also deep corneal lesion with corneal transplantation. Obviously, it has many advantages than HAM, 3T3, and CLs, especially in deep lamellar corneal disease. Zhu et al. and Du et al. report that APCS implants could be integrated with body tissues and used as seed carriers and sufficiently support new tissue regeneration and reconstruction [39, 40]. After successful transplantation, the cells will possibly differentiate to replace the damaged cells and restore the functions. So, the next step is operating corneal transplantation using COMECs on APCS in animal models.

## 5. Conclusion

We have established a novel technique for COMECs on the APCS using a feeder cell-free and serum-free culture environment, and transplantable tissue-engineered epithelial cell sheets are produced. The cultured cell sheets are morphologically and phenotypically retaining characters of stem cells. Animal trial using this technique has been initiated for the ocular surface reconstruction with both LSCD and deep corneal lesions.

## Figures and Tables

**Table 1:** Parameters for RT-PCR analysis of different genes in COMECs.

Name	Primer	Sequence	Product size (bp)
CK3	Forward	5′-ACCTGGGAAAGCACGAGAA-3′	120
	Reverse	5′-GGTCAGCGTTGGAGACATCA-3′	
ΔNP63*α*	Forward	5′-GAGGTTGGGCTGTTCATCAT-3′	188
	Reverse	5′-AGGAGATGAGAGGGGAGGA-3′	
ABCG2	Forward	5′-TGGTGCCCTTTACTTTGGTC-3′	234
	Reverse	5′-ACACTTGGCAAGAACCTCAT-3′	
